# Detection of clade types (clades I and II) within *Anopheles funestus sensu stricto* by the hydrolysis probe analysis (Taqman assay)

**DOI:** 10.1186/1756-3305-6-173

**Published:** 2013-06-12

**Authors:** Kwang Shik Choi, Maureen Coetzee, Lizette L Koekemoer

**Affiliations:** 1 Malaria Entomology Research Unit, School of Pathology, Faculty of Health Sciences, University of the Witwatersrand, Johannesburg, South Africa; 2 Centre for Opportunistic, Tropical and Hospital Infections, National Institute for Communicable Diseases, Division of the National Health Laboratory Service, Johannesburg, South Africa

**Keywords:** *Anopheles funestus*, Clades, Malawi, Mozambique

## Abstract

**Background:**

Recent studies presented two clades (clades I and II) within the major malaria vector, *Anopheles funestus s.s.* on the mitochondrial DNA. We describe a hydrolysis probe analysis (Taqman assay) method for the rapid identification of these two clades.

**Findings:**

A total of 53 *An. funestus s.s*. from Malawi and Mozambique were tested for detection of clade types using the hydrolysis probe analysis. Results were compared to DNA sequence analysis to verify the accuracy of the probes Taqman assay for this vector species. Analysis using the hydrolysis probe revealed that there were 21 individuals from Malawi and 13 individuals from Mozambique for clade I, and 19 individuals from Mozambique for clade II. The results were consistent with the results of DNA sequences. A field sample from northern Zambia revealed the presence of both clade types.

**Conclusion:**

A diagnostic method using the hydrolysis probe analysis was developed to identify clade types within *An. funestus s.s*. This assay will be useful for screening clade types of field-collected *An. funestus* specimens accurately and efficiently in malaria vector research and control studies.

## Findings

### Background

One of the major African malaria vectors, *Anopheles funestus* Giles belongs to a group of at least eleven species, all of which are morphologically similar at the adult stage [1-5]. It is widespread in sub-Saharan Africa including Madagascar [1,6] and is the most anthropophilic and endophilic species of the group [1]. Michel *et al*. [7] reported that the population structure of *An. funestus s.s.* based on *NADH Dehydrogenase subunit 5* (*ND5*) data revealed two cryptic subdivisions, clade I widespread throughout Africa and clade II known only from Mozambique and Madagascar. Choi *et al*. [8] also reported that *An. funestus s.s.* from Mozambique has two clades based on the DNA sequence analysis of *ND5* and *Cytochrome Oxidase I* (*COI*).

The hydrolysis probe analysis combines PCR amplification reaction and visualisation into a single step. This method uses oligonucleotide probes dual-labelling with a fluorescent reporter dye and a quencher molecule. The probe-specific product is amplified and this leads to cleavage of the probe, generating an increase in reporter fluorescence. The reporter dye is released away from the quencher and cleavage of allele-specific probes can be observed in a single PCR by using different reporter dyes [9,10]. The hydrolysis probe analysis can be considered to be one of the best methods with regard to specificity and sensitivity for detecting one substitution such as the knockdown resistance (*kdr*) mutations [10,11].

In this article, we report on the use of a hydrolysis probe assay (Taqman assay) that is able to accurately and rapidly identify the two clade types of *An. funestus s.s.*

## Methods

### Mosquito sample collection and identification

Specimens were collected resting inside houses from Karonga (10°19′S, 34°08′E) and Nkhota kota (12°55′S, 34°18′E) in Malawi, Chibuto (24°40′S, 33°33′E) in Mozambique and Nchelenge (9°21′S, 28°44′E) in Zambia. The samples were identified using the morphological keys of Gillies and Coetzee [2] and the molecular method of Koekemoer *et al. *[12]. All DNA samples were extracted from either single mosquitoes or available parts of mosquitoes using the Ballinger-Crabtree protocol [13]. The DNA templates were resuspended in TE buffer at volumes between 50 μL and 100 μL.

### Hydrolysis probe analysis

PCR was performed using a CFX96™ Real-Time system (Bio-Rad). The primers and probes were designed from *COI*. The sequence information of *COI* was presented in Choi *et al*. [8]. Two standard oligonucleotides (MACROGEN) and two minor groove binding (MGB) probes (Applied Biosystems) were used. Primers Forward (5′- GCA GGA ACA GGA TGA ACA GT -3′) and Reverse (5′- GAA ATT CCT GCT AAA TGT AAT GAA A -3′) were used for binding the flanking region of both clade mutation sites in the *COI*. The probe clade I (5′- TCA GGA ATT GCT CAT GCT -3′) was labelled with 6-FAM for the detection of the clade I and the probe clade II (5′- TCA GGA ATT GCC CAT GCT -3′) were labelled with VIC for detection of the clade II. The 20 μL PCR reaction contained 1 μL of the genomic DNA of an individual mosquito, 10 μL of IQ™ Supermix (Bio-Rad), 0.8 μM of each primer and 0.4 μM of each probe. The PCR cycling conditions were as follows: an initial denaturation at 95°C for 10 minute, followed by 45 cycles of 95°C for 10 seconds and 63°C for 45 seconds. The increase in FAM and VIC fluorescence was monitored in real time by detecting fluorescence on the yellow channel (450–490 nm excitation and 515–530 nm detection) for clade I and the blue (515-535 nm excitation and 560-580 nm detection) for clade II. The Malawi and Mozambique results were confirmed with the sequence results to ensure that experimental error was limited.

## Results and discussion

**Figure 1:**
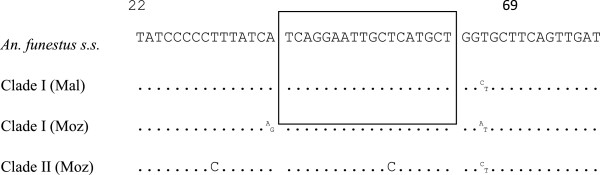
**Alignment from 3′ to 5′ end of the COI target region for probes.** The alignment of *An. funestus* for the comparison were originated from GenBank (access No. AY423059). Mal and Moz represent Malawi and Mozambique respectively. Dots represent identity with respect to the *An. funestus* sequences. ^A^_G_, ^C^_T_ and ^A^_T_ indicate polymorphic positions. The square indicates the sequences of the probes for clades I and II.

**Figure 2:**
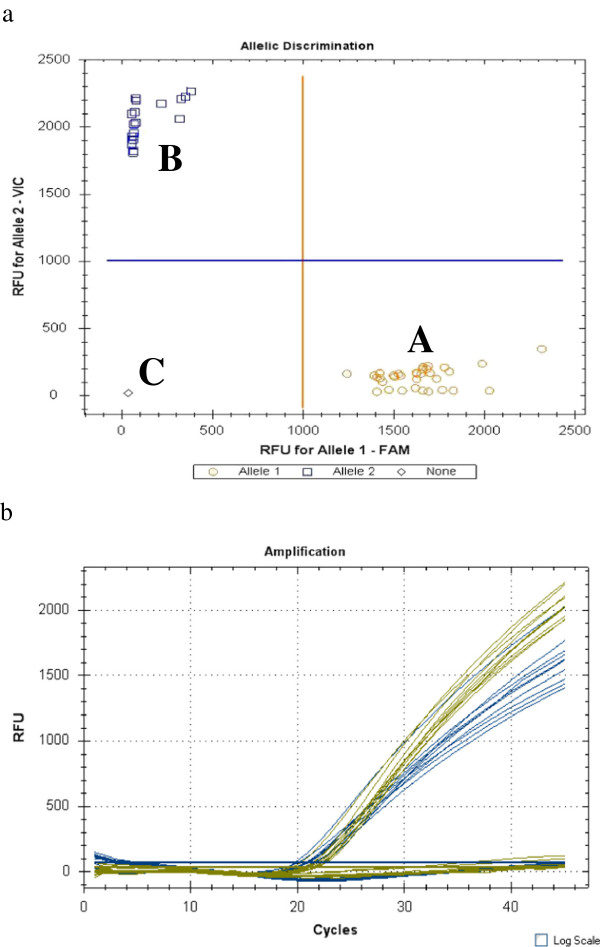
**Hydrolysis probe analysis fluorescence results for clades I and II of *An. funestus s.s. *from Malawi and Mozambique.** Scatter plot analysis (**a**) of **A**) clade I; **B**) clade II; **C**) negative control and quantitative peaks (**b**). Blue peaks are clade I and yellow peaks are clade II.

**Figure 3:**
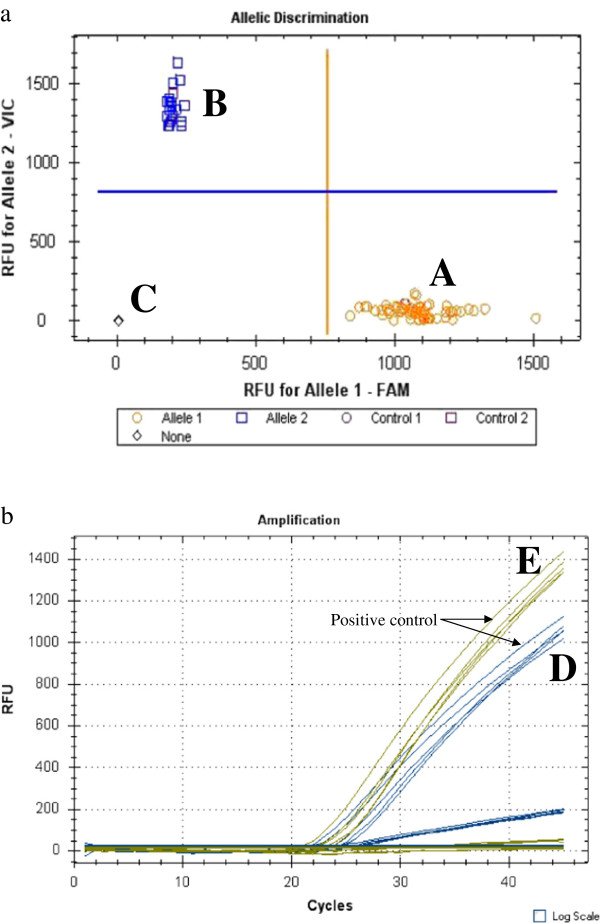
**Hydrolysis probe analysis fluorescence results for clades I and II of *An. funestus s.s. *from Zambia.** Scatter plot analysis **(a)** and quantitative peaks **(b)** of **A**) clade I; **B**) clade II; **C**) negative control. Blue peaks are clade I and yellow peaks are clade II. Positive controls (arrows) from the Figure 2 experiment are **D**) clade I and **E**) clade II.

A total of 53 specimens were used for the assay, 21 from Malawi and 32 from Mozambique. The two specific probes for clades I and II were designed based on one substitution between clades in the partial region of *COI* (Figure 1). The first specific probe for the clade I allele was labelled with FAM and the second specific probe for the clade II allele was labelled with VIC. All 21 *An. funestus* individuals from Malawi were clade I type while 13 and 19 individuals from Mozambique were clades I and II respectively (Figure 2). The results from the hydrolysis probe analysis in this study were 100% consistent with the results from the DNA sequencing. Subsequently, a field sample of 78 *An. funestus* individuals from Nchelenge in northern Zambia became available and were successfully analysed as clades I (n = 59) and II (n = 19), using the above sequenced specimens as positive controls (Figure 3).

This assay will save time when routinely identifying collections of wild mosquitoes belonging to this important malaria vector species. Although this method is expensive compared to the PCR method, it is much more accurate than PCR, cheaper than DNA sequence analysis, and avoids the potential safety hazard presented by the use of ethidium bromide [10]. Similar results were reported by Bass *et al*. [10] who developed the hydrolysis probe analysis (Taqman assay) for detection of the *kdr* mutations in *Anopheles gambiae* based on one nucleotide substitution, concluding that it is the most specific and sensitive method compared with seven other diagnostic assays including the PCR method.

At present we do not know if the various molecular and chromosomal forms in *An. funestus* are associated with insecticide resistance or vector competence, nor whether “intergrades” can be found in different populations. It is also not clear whether the RFLP types [14], the clades [7] and the chromosomal forms [15] in *An. funestus* are themselves in any way associated. Since *An. funestus* is such an important malaria vector in Africa, it is vital that we understand the basic underlying genetics of the species as this will allow us to better comprehend its role in malaria transmission and the evolution and spread of insecticide resistance.

## Conclusion

The application of the assay described here is expected to greatly improve the efficiency of screening large-scale field-collected samples of *An. funestus s.s.* for clades I and II. Hence, this assay could help elucidate the role that each clade is playing in malaria transmission and whether there is any difference in insecticide resistance frequencies.

## Competing interests

The authors declare no competing interests.

## Authors’ contributions

KSC designed the study, developed the new hydrolysis probe analysis and drafted the manuscript. LLK and MC assisted with analysis of the data and helped draft the manuscript. All authors read and approved the final manuscript.

## References

[B1] Gillies MT de Meillon B The Anophelinae of Africa South of the Sahara 1968 Johannesburg, South: Publications of the South African Institute for Medical Research

[B2] Gillies MT Coetzee M A Supplement to the Anophelinae of Africa South of the Sahara (Afrotropical Region) 1987 Johannesburg, South Africa: Publications of the South African Institute for Medical Research

[B3] Couhet A Simard F Toto J-C Kengne P Coetzee M Fontenille D Species identification within the *Anopheles funestus* group of malaria vectors in Cameroon and evidence for a new species Am J Trop Med Hyg 2003 69 200 205 13677376

[B4] Harbach RE The classification of genus *Anopheles* (Diptera: Culicidae): a working hypothesis of phylogenetic relationships Bull Entomol Res 2004 94 537 553 15541193 10.1079/ber2004321

[B5] Spillings BL Brooke BD Koekemoer LL Chiphwanya J Coetzee M Hunt RH A new species concealed by *Anopheles funestus* Giles, a major malaria vector in Africa Am J Trop Med Hyg 2009 81 510 515 19706923

[B6] Mouchet J Manguin S Sircoulon J Laventure S Faye O Onapa AW Carnevale P Julvez J Fontenille D Evolution of malaria in Africa for the past 40 years: impact of climatic and human factors J Am Mosq Control Assoc 1998 14 121 130 9673911

[B7] Michel AP Ingrasci MJ Schemerhorn BJ Kern M Le Goff G Coetzee M Elissa N Fontenille D Vulule J Lehmann T Sagnon N Costantini C Besansky NJ Rangewide population genetic structure of the African malaria vector *Anopheles funestus* Mol Ecol 2005 14 4235 4248 10.1111/j.1365-294X.2005.02754.x 16313589

[B8] Choi KS Koekemoer LL Coetzee M Population genetic structure of the major malaria vector *An. funestus s.s.* and allied species in southern Africa Parasit Vectors 2012 5 283 10.1186/1756-3305-5-283 23216696 PMC3533957

[B9] Livak KJ Allelic discrimination using fluorogenic probes and the 5′ nuclease assay Genetic Anal 1999 14 143 149 10.1016/S1050-3862(98)00019-9 10084106

[B10] Bass C Nikou D Donnelly MJ Williamson MS Ranson H Ball A Vontas J Field L Detection of knockdown resistance (*kdr*) mutations in *Anopheles gambiae:* a comparison of two new high-throughput assays with existing methods Malaria J 2007 6 111 10.1186/1475-2875-6-111 PMC1971715 17697325

[B11] Choi KS Spillings BL Coetzee M Hunt RH Koekemoer LL A comparison of DNA sequencing and the hydrolysis probe analysis (TaqMan assay) for knockdown resistance (*kdr*) mutations in *Anopheles gambiae* from the Republic of the Congo Malaria J 2010 9 278 10.1186/1475-2875-9-278 PMC2959077 20937156

[B12] Koekemoer LL Kamau L Hunt RH Coetzee M A cocktail polymerase chain reaction assay to identify members of the *Anopheles funestus* (Diptera: Culicidae) group Am J Trop Med Hyg 2002 66 804 811 12224596 10.4269/ajtmh.2002.66.804

[B13] Ballinger-Crabtree ME Black WC Miller BR Use of genetic polymorphisms detected by the Random-Amplified Polymorphic DNA Polymerase Chain Reaction (RAPD-PCR) for differentiation and identification of *Aedes aegypti* subspecies and populations Am J Trop Med Hyg 1992 47 893 901 1471745 10.4269/ajtmh.1992.47.893

[B14] Koekemoer LL Kamau L Garros C Manguin S Hunt RH Coetzee M Impact of the Rift Valley on Restriction Fragment Length Polymorphism Typing of the Major African Malaria Vector *Anopheles funestus* (Diptera: Culicidae) J Med Entomol 2006 43 1178 1184 10.1603/0022-2585(2006)43[1178:IOTRVO]2.0.CO;2 17162950

[B15] Costantini C Sagnon NF Ilboudo-Sanogo E Coluzzi M Boccolini D Chromosomal and bionomic heterogeneities suggest incipient speciation in *Anopheles funestus* from Burkina Faso Parassitologia 1999 41 595 611 10870569

